# What does it Mean to be a Good Mentor?

**DOI:** 10.1055/a-2440-2332

**Published:** 2024-11-13

**Authors:** Joon Pio Hong

**Affiliations:** 1 Department of Plastic and Reconstructive Surgery, Asan Medical Center, University of Ulsan College of Medicine, Seoul, Republic of Korea

**Figure:**
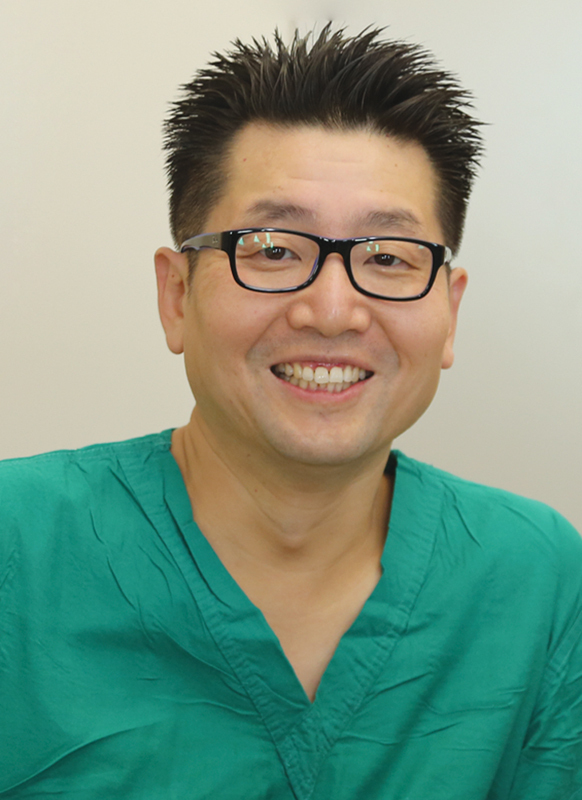
Joon Pio Hong: Editor-in-Chief

According to the Merriam-Webster dictionary, the word “mentor” is defined as a trusted counselor or a guide. The word “mentor” comes from Greek mythology. In the Odyssey, Mentor was the son of Alcimus. In his old age, Mentor was a friend of Odysseus. When Odysseus left for the Trojan War, he placed Mentor in charge of his son Telemachus, and of Odysseus' palace. Ultimately, the term was adopted in Latin and other languages meaning someone who imparts wisdom to and shares knowledge with a less-experienced colleague. From this story, one can assume that this word is deeply rooted in trust, wisdom, and teaching. So what does mentoring mean in the modern world and what is being a good mentor?

First is wisdom which is obtained from knowledge, skills, dedication, and most of all experience. Thus a mentor is able to guide and counsel the mentee based on his wisdom. 1 2 Second is teaching, passing down his or her knowledge allowing them to reach their full potential. These two attributes are often well-delivered helping the mentees grow. However, it is the third attribute, “trust,” that is challenging and often not achieved enough. Trust is bound by authentic personal relationships rather than professional relationships. Trust is about empathy and compassion, having the mentee know that you really care about them. Trust is about logic when the mentee builds faith in the mentor's judgement. 3 4 It is about telling the mentee that you understand, it is about telling the mentee that you can do it, and it is about really wanting the mentee to succeed even surpassing the mentor yourself. The mentor must be committed to the growth and success of the mentee. It not only requires time but energy, resources, and most of all sincere friendship. The mentor must understand the mentee's goals, aspirations, and challenges to help them navigate throughout the journey. It begins with actively and empathetically listening while holding judgement allowing them to share what is deep down inside the mentee's mind. Carefully sharing constructive criticism with the thought focused on the mentee's success. This is why when someone asks who is your mentor, it sometimes is difficult to identify a true mentor in one's life.

When you embark on the journey of mentoring someone, make sure you do it in a way that impacts the mentee in a meaningful way. It will be difficult but when you do it right, a good mentor can make a positive impact on their mentee's personal and professional development, helping them achieve their goals and reach their full potential. Most of all you will have a friend for life that transcends time, age, and wisdom.

So you can be the “mentor” from Odyssey, the man who was a supporter, counselor, teacher, advisor, and most of all a friend to Telemachus.
